# A New Ultrasensitive Bioluminescence-Based Method for Assaying Monoacylglycerol Lipase

**DOI:** 10.3390/ijms22116148

**Published:** 2021-06-07

**Authors:** Matteo Miceli, Silvana Casati, Pietro Allevi, Silvia Berra, Roberta Ottria, Paola Rota, Bruce R. Branchini, Pierangela Ciuffreda

**Affiliations:** 1 Dipartimento di Scienze Biomediche e Cliniche “Luigi Sacco”, Università degli Studi di Milano, Via G.B. Grassi 74, 20157 Milano, Italy; matteo.miceli@unimi.it (M.M.); silvana.casati@unimi.it (S.C.); silvia.berra@unimi.it (S.B.); roberta.ottria@unimi.it (R.O.); 2 Dipartimento di Scienze Biomediche, Chirurgiche e Odontoiatriche, Università degli Studi di Milano, Via della Commenda 10, 20122 Milano, Italy; pietro.allevi@unimi.it (P.A.); paola.rota@unimi.it (P.R.); 3 Department of Chemistry, Connecticut College, New London, CT 06320, USA; brbra@conncoll.edu

**Keywords:** bioluminescence, monoacylglycerol lipase, kinetic assay, PLG2

## Abstract

A novel bioluminescent Monoacylglycerol lipase (MAGL) substrate 6-O-arachidonoylluciferin, a D-luciferin derivative, was synthesized, physico-chemically characterized, and used as highly sensitive substrate for MAGL in an assay developed for this purpose. We present here a new method based on the enzymatic cleavage of arachidonic acid with luciferin release using human Monoacylglycerol lipase (*h*MAGL) followed by its reaction with a chimeric luciferase, PLG2, to produce bioluminescence. Enzymatic cleavage of the new substrate by MAGL was demonstrated, and kinetic constants Km and Vmax were determined. 6-O-arachidonoylluciferin has proved to be a highly sensitive substrate for MAGL. The bioluminescence assay (LOD 90 pM, LOQ 300 pM) is much more sensitive and should suffer fewer biological interferences in cells lysate applications than typical fluorometric methods. The assay was validated for the identification and characterization of MAGL modulators using the well-known MAGL inhibitor JZL184. The use of PLG2 displaying distinct bioluminescence color and kinetics may offer a highly desirable opportunity to extend the range of applications to cell-based assays.

## 1. Introduction

In the last decade, the endocannabinoid system (ECS) has stimulated renewed interest, thanks to medical research results based on modern technologies highlighting its large distribution in human tissues [1,2] and its involvement in several physiological and pathological processes [3]. Indeed, deeper investigations in this field have demonstrated how the dysregulation of the ECS can induce pathological conditions such as pain and inflammation [4,5,6], neurological disorders [7], and cancer [8,9,10]. Although cannabinoid receptor stimulation [11,12] and endocannabinoid turnover modulation [13,14,15] have been investigated, targeting the ECS through enzyme modulation remains an ambitious objective [16]. In this regard, several studies have shown the involvement of the monoacylglycerol lipase (MAGL) as a playmaker in simultaneously coordinating multiple lipid signaling pathways in both physiological and disease contexts [17], thus suggesting a role as a therapeutic target.

MAGL inhibitors are potential antiproliferative and cytostatic drugs for cancer chemotherapy not only for enhancing endocannabinoid signaling but also controlling fatty acid release for synthesis of protumorigenic signaling lipids. Moreover, these inhibitors are also antineurodegenerative compounds that induce an anti-inflammatory activity by reducing the production of eicosanoids. The role of MAGL as a therapeutic target has increased interest not only in the development of selective inhibitors but also in the set-up of efficient protocols amenable to high-throughput screening (HTS) formats. In this context, different analytical methods to determine MAGL activity, generally based on its enzymatic action on fatty acid compounds, have been developed using various techniques. The procedures most used to screen MAGL inhibitors quantify the arachidonic acid released from 2-AG hydrolysis using high-performance liquid chromatography (HPLC) coupled with UV [18] or mass spectrometric detector [19]. Furthermore, a fluorescence-based assay employing 7-hydroxycoumarinyl arachidonate (7-HCA) substrate [20] and new procedures for MAGL continuous assay based on long-wavelength fluorogenic substrates, 7-hydroxyresorufinyl arachidonate [21] and 7-hydroxyresorufinyl octanoate [22] have been reported.

Bioluminescence (BL) approaches offer several advantages such as high sensitivity, lowering of the background, wide dynamic range, and relative ease of performance. Beetle luciferases have been extensively investigated and are being widely used for bioanalytical purposes, and many of these applications can be used in a high-throughput screening format in support of drug development [23]. Luciferases catalyze the ATP-dependent oxidation of firefly D-luciferin (LH_2_), a benzothiazole-containing compound, in a two-step reaction [24]: (i) adenylation of LH_2_ with ATP, producing luciferyl adenylate with the release of pyrophosphate, and (ii) oxidation of the luciferyl adenylate by molecular oxygen, producing a dioxetanone that rapidly cleaves, generating CO_2_ and electronically excited singlet oxyluciferin, which decays by emitting a photon of light in the green to the red range with a quantum yield of 15–60%, depending on the luciferase [25,26]. Bioluminescent imaging (BLI) has been employed for imaging various biological phenomena, such as protein–protein interaction [27] and gene regulation [28]. The firefly luciferase system is widely used for BLI systems, which generally employ LH_2_ or aminoluciferin as a substrate in the presence of ATP, Mg^2+^, and O_2_ to produce detectable photons. Based on this strategy, several bioluminescent probes have been applied to the in vivo imaging of H_2_S, F^−^, H_2_O_2_, nitroreductase, aminopeptidase, and so on [29,30,31,32,33].

**Scheme 1:**
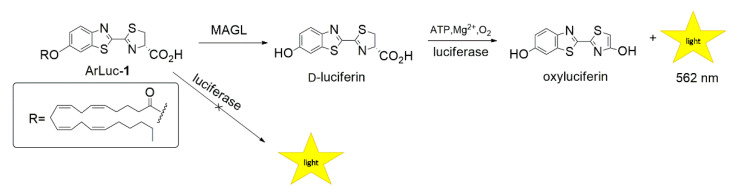
Bioluminescence reactions of compound 6′-O-arachidonoylluciferin (ArLuc-**1**). The substrate chemical structure and biochemical reactions leading to 562 nm peak light emission (λ max) are shown.

Given our continued interest in the development of selective probes for MAGL suitable for in vitro and in vivo assays, we report here a straightforward assay in which arachidonoyl luciferin (ArLuc-1) is hydrolyzed by MAGL, and the released LH_2_ reacts with PLG2 to generate light in a single reaction mixture (Scheme 1). LH_2_ was chosen as the substrate to be modified through an ester linkage with the arachidonoyl group, because it produces readily detected (with PMTs) 562 nm maximum BL.

Indeed, it has been found that the 6′-hydroxy group of LH_2_ is crucial for BL [34]; therefore, the blocking of this function essentially quenches BL. Once the caged substrate is cleaved by MAGL, BL can be produced via the reaction with luciferase (Scheme 1).

PLG2 was used instead of the wild-type enzyme, because it is a thermostable luciferase that produces a bright BL signal and is resistant to low pH shifting, making it well suited for cell-based assays.

## 2. Results and Discussion

### 2.1. Design and Synthesis of the Probe

**Scheme 2:**
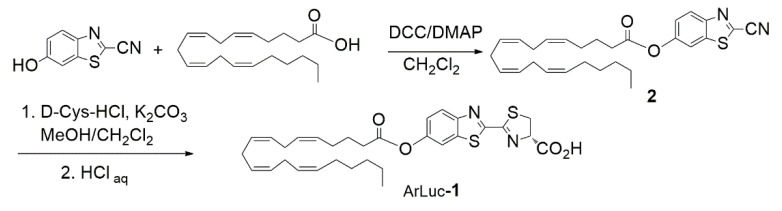
Chemical synthesis of ArLuc-**1**.

In this study, a novel BL assay for MAGL was established. To resolve the optimum BL assay conditions for MAGL using the luciferase–luciferin reaction, the substrate ArLuc-**1** was designed, and various conditions for the assay were examined. The instability of LH_2_ toward acid, base, light, and oxygen [35], together with the presence of the carboxyl group, precluded direct esterification of LH_2_ at the 6′-position. However, LH_2_ is stable in solution at neutral pH, whereas in acid solution (pH < 2), a rapid degradation to D-cysteine, and a benzothiazole derivative occurs. In alkaline solution, LH_2_ is oxidized to dehydroluciferin, which is a potent inhibitor of luciferase [36]. Consequently, the ester of LH_2_ is prepared, as in the synthesis of other analogues, by construction of the labile thiazoline ring at a late stage in the synthesis. The synthesis of ArLuc-**1** (Scheme 2) follows a variation of the method applicable to the syntheses of LH_2_ derivatives modified at the 6′-position [37]. *N*,*N*′-dicyclohexylcarbodiimide (DCC) and 4-dimethylaminopyridine (DMAP) were used to directly couple arachidonic acid to the commercially available 6-hydroxy-2-cyanobenzothiazol, producing compound 2 in nearly quantitative yield. Compound 2 was then reacted with D-cysteine hydrochloride under conditions that did not hydrolyze the ester yielding ArLuc-**1** in 52% overall yield. ArLuc-**1** was completely characterized by ^1^H– and ^13^C–NMR and 2D–COSY, HMQC, and HMBC correlation spectra. The purity, assessed by H1–NMR, was greater than 98%, and no signals for free LH_2_ were detected. The purity of the compound was also verified by thin layer chromatography and by luminometric assay.

**Figure 1:**
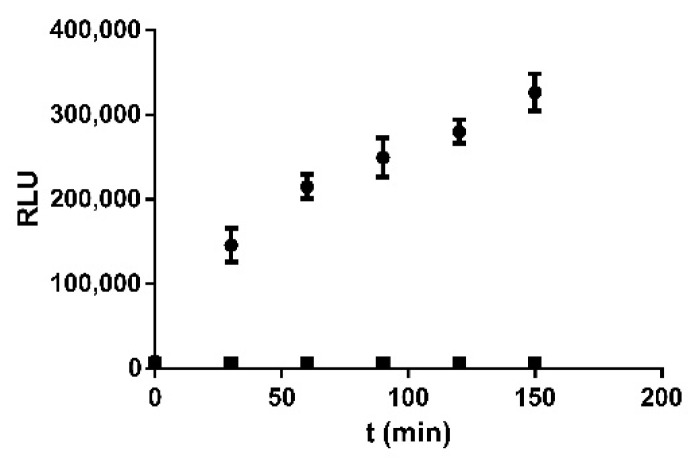
Comparison of luminescence produced by ArLuc-**1** in presence (●) or absence (▪) of Monoacylglycerol lipase (MAGL). Data represent mean ± standard deviation (SD).

Because of the high sensitivity of the luminometric assay [38], the luciferin derivative had to be free of LH_2_, since traces of it in substrate preparations would result in a significant increase of background luminescence and lower the sensitivity of the method. To ensure that the observed BL signal was due only to the consecutive hydrolysis and PLG2 reactions and assess the stability of the substrate in reaction condition, the enzymatic assay was further investigated by systematic elimination of MAGL from the assay. The results (Figure 1) show that LH_2_ production is not observed unless ArLuc-**1** is hydrolyzed by MAGL, demonstrating the stability of the substrate under assay condition.

### 2.2. PLG2 Expression and Purification

We expressed PLG2, a thermostable and specific activity enhanced green (*λ*max = 559 nm) light-emitting luciferase that was engineered from a chimeric protein consisting of the large N-terminal domain of *P. pyralis* luciferase fused to the small C-terminal domain of *Luciola italica* luciferase [39]. Because PLG2 is a thermostable luciferase that produces a bright BL signal and is resistant to low pH shifting, it is well suited for cell-based assays. *E. coli* JM109 competent cells were transformed with the pQE-30 vector containing the PLG2 cDNA sequence fused to an N-terminal 6xHis tag. After expression, PLG2 was purified by affinity chromatography and obtained in 28.7 mg/L yield [24].

### 2.3. PLG2 Activity: Effects of DMSO and ArLuc-***1***

**Figure 2:**
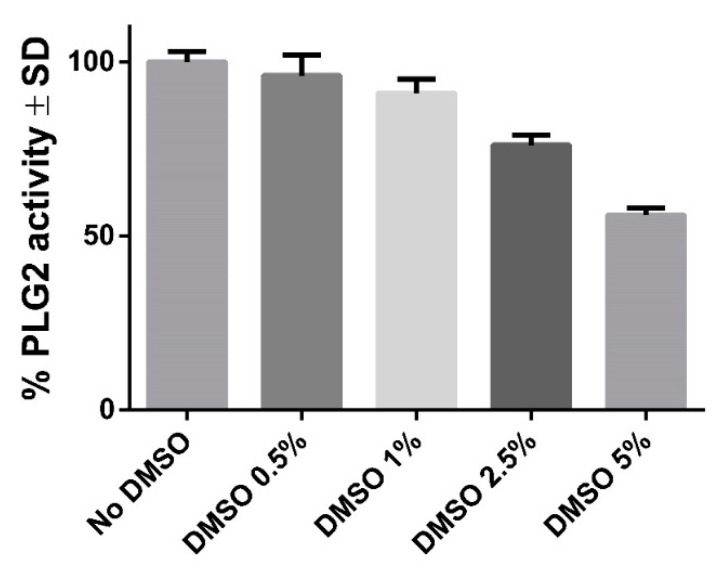
PLG2 luminescence emission in the presence of varying concentrations from 0.5 to 5% of DMSO. Error bars represent mean ± SD.

To develop the bioluminescence assay, several preliminary controls were performed. The effect of DMSO was investigated to determine whether the solvent would influence the assay readout. DMSO is commonly used to solubilize substrates and inhibitors that are poorly soluble in aqueous solutions in enzymatic assays, but it can inhibit some enzymes in a concentration-dependent manner. In fact, while luciferases exhibit high selectivity and sensitivity, practical applications can be limited by low resistance to organic solvents. Several concentrations of DMSO (0.5%, 1%, 2.5%, and 5%) were assessed to determine sufficient ArLuc-**1** solubility levels that did not inhibit PLG2 activity. As shown in Figure 2, 2.5% (*v*/*v*) DMSO is a good compromise between the complete solubilization of the substrate and a tolerable reduction in PLG2 activity.

**Figure 3:**
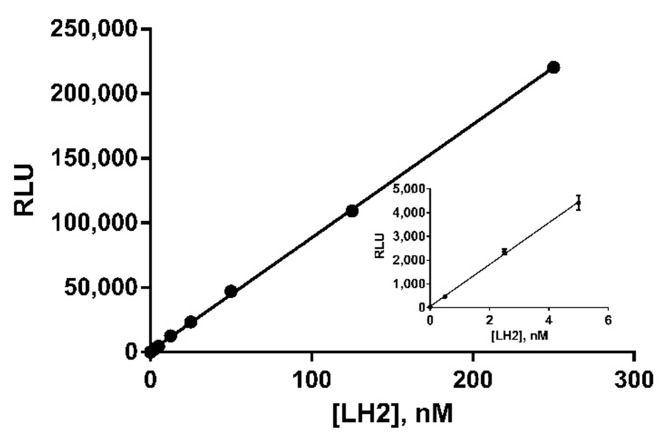
Luminescence (RLU) of PLG2 as a linear function of LH_2_ concentration in the presence of 2.5% DMSO. All points are expressed as mean ± SD.

Next, the assay required a standard curve relating relative light units (RLU) to the concentration of LH_2_ produced (Figure 3). Calibration curves with LH_2_ standards (0 to 250 nM) were made for PLG2, as described in Materials and Methods, and linearity was observed over the entire concentration range tested.

**Figure 4:**
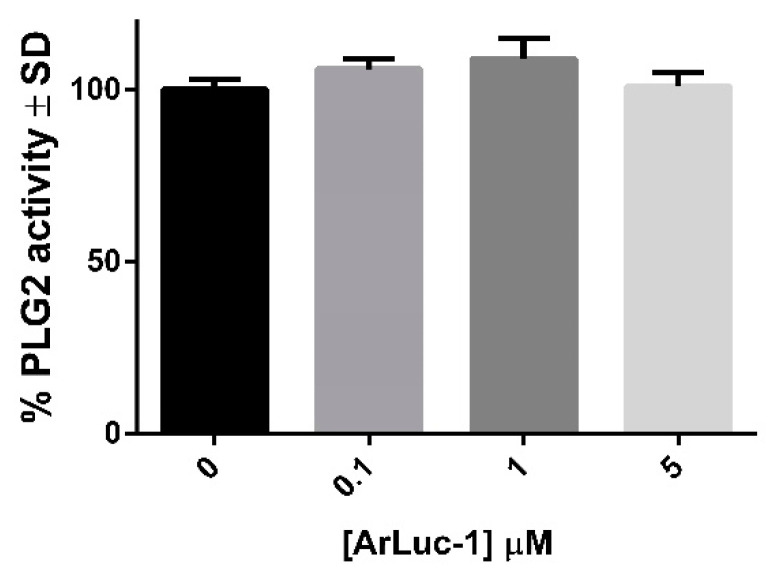
PLG2 activity in presence of the indicated concentrations of ArLuc-1. Error bars represent mean ± SD.

The limit of detection (LOD) and limit of quantification (LOQ) of the assay were calculated from calibration curves and blank values according to the formulas LOD = 3.3σ/s and LOQ = 10σ/s, where “σ” is the averaged standard deviation of the blanks and “s” is the slope of the calibration curve [40,41]. The resulting values were 0.09 nM for LOD and 0.3 nM for LOQ. Subsequently, ArLuc-**1** was evaluated as a potential inhibitor of PLG2, because the absence of this interference is essential for the sensitivity of the assay. To address this issue, luminescence experiments were performed with PLG2 and LH_2_ in the presence of increasing concentrations of ArLuc-**1** (0.1, 1, 5 µM). Reassuringly, PLG2 activity is essentially independent of ArLuc-**1** at the concentrations tested (Figure 4).

### 2.4. ArLuc-***1*** as MAGL Substrate: Assay Development and Validation

**Figure 5:**
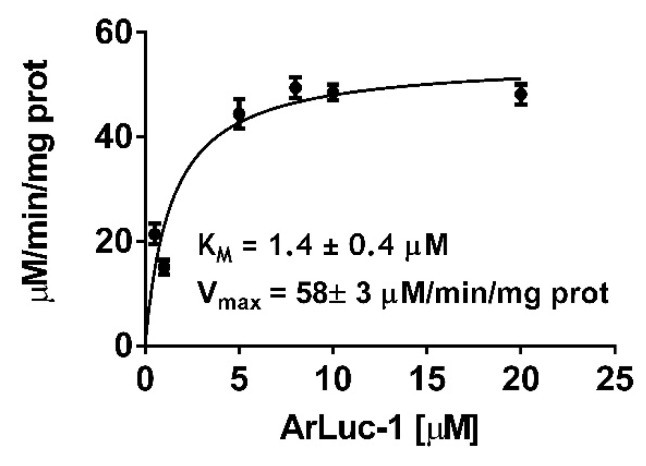
Kinetic study of MAGL activity with ArLuc-**1**. Data are reported as mean ± SD. The curve model is that of the Michaelis–Menten.

After demonstrating excellent sensitivity and linearity for LH_2_ detection and establishing that MAGL could hydrolyze ArLuc-**1** and release free LH_2_ (Figure 1), the two-step MAGL assay was further investigated by steady state kinetics analysis. Briefly, 10 μL of *h*MAGL enzyme solution (25 ng) were pipetted into the wells of a white 96-well plate, and 90 μL of PLG2 assay mix, containing varying amounts of ArLuc-**1** (final concentrations 0.5, 1, 5, 8, 10, and 20 μM) were quickly added by automatic injector. Luminescence was read immediately and at 3 min intervals for 10 min. Control experiments yielded a very low background (Figure 1) signal with ArLuc-**1** in the absence of MAGL. Measurement of the net luminescent signal at varying concentrations of substrate ArLuc-**1** were converted into amounts of LH_2_ produced using calibration curves. The data were fit to the Michaelis–Menten equation to obtain kinetic parameters Km and Vmax (Figure 5).

**Figure 6:**
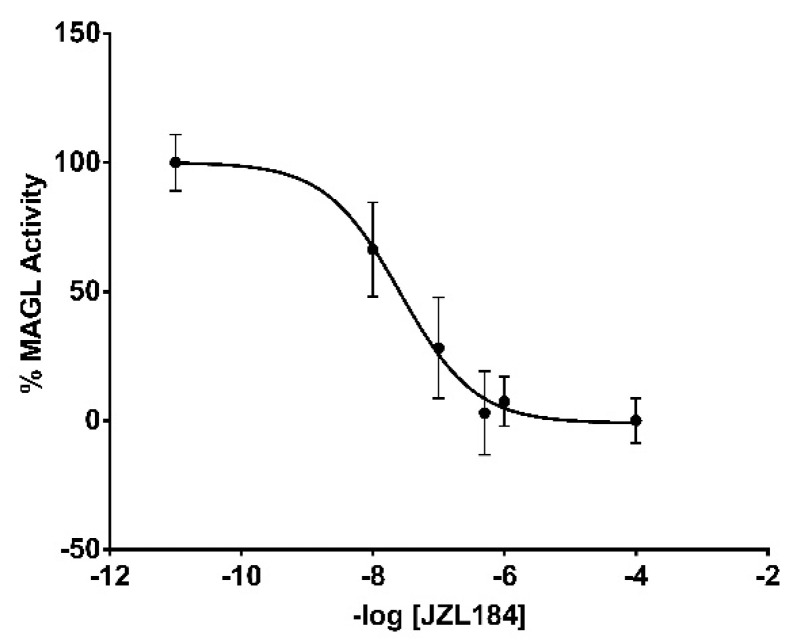
Concentration-dependent inhibition of JZL184 on MAGL. Data, reported as mean ± SD, are expressed as the percentage of controls (without JZL184).

The final step in the validation of the assay was undertaken with JZL184, a known MAGL inhibitor [42], to substantiate that ArLuc-**1** is a true substrate for MAGL. DMSO solutions of JZL184 (100, 1, 0.5, 0.1, and 0.01 μM) were incubated with hMAGL for 20 min, and then the results of subsequent two-step assays were plotted as a dose-response curve (Figure 6). The activity of *h*MAGL was calculated as described for the kinetic assay. The IC50 for JZL184 was found to be 260 nM, which agrees well with values reported by Lauria et al. (217 nM) and by Savinainen et al. (209 nM) [21,43].

### 2.5. Docking Studies

We also performed a docking study of ArLuc-**1** using a crystallographic model the structure of human monoacylglycerol lipase (*h*MAGL) co-crystallized in complex with a noncovalently bound inhibitor (PDB ID: 5ZUN, resolution 1.35 Å). This structure recently was effectively used in a structure-based drug design of a novel series of reversible MAGL inhibitors [44]. The docking procedure (see experimental) was validated by docking the cognate ligand (1-(2-chlorobiphenyl-3-yl)-4-[4-(1,3-thiazol-2-ylcarbonyl)-piperazin-1-yl]pyrrolidin-2-one) of 5ZUN. This ligand was built through the Schrödinger Maestro Build Toolbar, prepared with Schrödinger Ligand Preparation, and docked into the apo form of 5ZUN protein. The obtained top pose (XP docking score −12.37 kcal mol-1) showed an excellent superimposition to its native crystallographic pose in 5ZUN (RMSD 0.16, maximum atomic displacement: 0.33 Å), confirming the accuracy of this approach.

**Figure 7:**
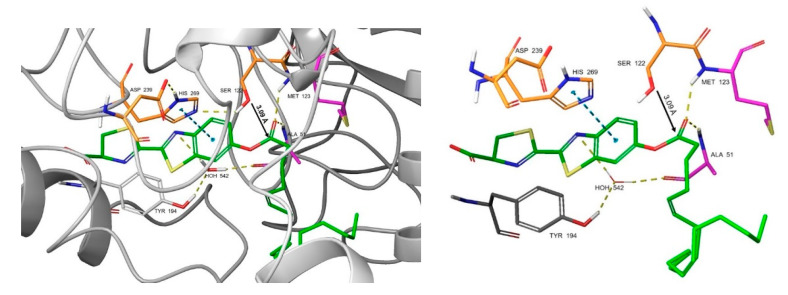
Top scoring docking pose of compound ArLuc-**1** (green stick) into the MAGL binding site. Residues are numbered according to the PDB crystal (5ZUN) convention. The triad catalytic residues are shown in orange sticks, those forming the oxyanion hole in magenta sticks. H-bonds and the π–π stacking interaction are represented with yellow and blue dashed lines, respectively. The black arrow represents the distance between the oxygen atom of the hydroxyl group of Ser122 and the carbon of the carbonyl group of ArLuc-**1**.

The top pose obtained by Glide docking of ArLuc-**1** shows a G score and MM–GBSA binding energy values (−10.33 and −78.68 kcal mol-1, respectively) that confirm its ability to bind to MAGL in the catalytic site. In particular, (Figure 7), the carbonyl oxygen atom of the substrate ester moiety is H-bonded to main chain nitrogen atoms of Ala51 and Met123. During catalysis, these residues constitute the oxyanion hole involved in the stabilization of the developing negative charge on the carbonyl in the transition state en route to the tetrahedral intermediate [45]. Moreover, the distance from the hydroxyl oxygen of the catalytic serine (Ser122) to the reactive carbonyl carbon of the substrate ester is 3.09 Å, which is the ideal position for the initial nucleophilic attack to take place.

**Figure 8:**
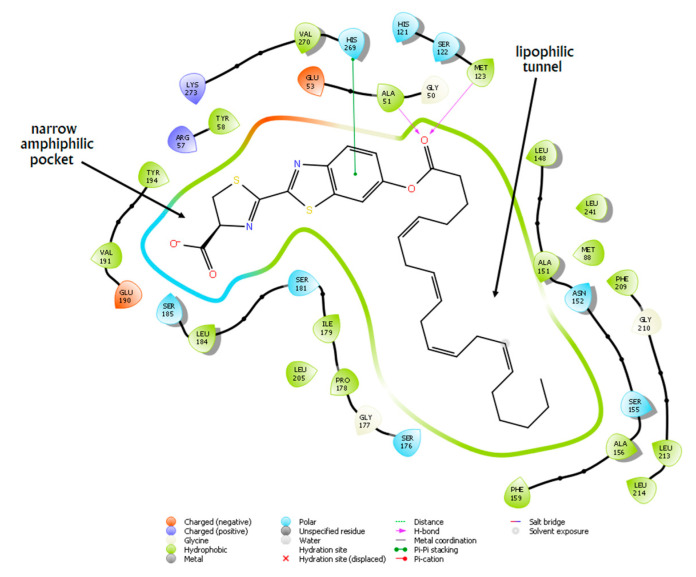
2D representation of interactions between MAGL binding site and ArLuc-**1**.

The luciferin moiety of ArLuc-**1** falls into the narrow amphiphilic pocket [44] of MAGL, and its benzothiazole component forms a π–π stacking interaction with the histidine of the catalytic triad (Ser122, His269, Asp239) [44], while its nitrogen atom is involved in a water-mediated H-bonding network with the Tyr194 hydroxyl group and the Ala51 main-chain carbonyl oxygen. Additionally, the arachidonoyl chain is well-accommodated in the large, hydrophobic pocket of MAGL (Figure 8) surrounded by hydrophobic/aromatic residues as Phe159, Phe209, Ala151, Ala156, Ile179, Leu148, Leu205, Leu213, Leu214, and Leu241. The extensive hydrophobic interactions can play a significant role in the stabilization of the complex.

## 3. Materials and Methods

### 3.1. Chemicals and Reagents

Reactions progress was monitored by analytical thin-layer chromatography (TLC) on precoated aluminum foil (Silica Gel 60 F254-plate, Sigma–Aldrich, St. Louis, MO, USA), and the products were visualized by UV light. Purity of all compounds (>98%) was verified by thin layer chromatography and NMR measurements [46,47]. The chemicals required for validation, all of analytical grade, and all reagents required for bacterial cell culture and the kit for plasmid extraction were purchased from Sigma–Aldrich (St. Louis, MO). *E. coli* JM109 competent cells were from Promega, (Fitchburg, WI, USA). Ni-NTA agarose and 3–12 mL 10kDa Slide-A-Lyzer™ Dialysis Cassette used for protein purification were purchased from Thermo Fisher Scientific (Waltham, MA, USA). SDS-PAGE experiments were performed using Mini-PROTEAN^®^ II handcast systems (Bio-Rad, Hercules, CA, USA) kits for both equipment and reagents, unless specified. Monoacylglycerol lipase (human recombinant, 50 μg, *h*MAGL) was purchased from Cayman Chemical (Ann Arbor, MI, USA). For bioluminescence experiments, LH_2_ potassium salt was purchased from Promega Italia Srl, while ATP magnesium salt was from Sigma–Aldrich.

### 3.2. Instruments

1H–NMR spectra were recorded in CDCl3 (isotopic enrichment 99.95%) solutions at 300 K using a Bruker AVANCE 500 instrument (500.13 MHz for 1H, 125.76 MHz for 13C) using 5 mm inverse detection broadband probes and deuterium lock. Chemical shifts (δ) are given as parts per million relative to the residual solvent peak (7.26 ppm for 1H and 77.0, central line, for 13C) and coupling constants (J) are in Hertz. For two-dimensional experiments (COSY-45, HSQC, and HMBC), standard Bruker microprograms using gradient selection (gs) were applied (for more information, see electronic Supplemental Data).

The quantification of the cDNA plasmid was done with a NanoDrop™ 2000 Microvolume Spectrophotometer (Thermo Fisher Scientific, Waltham, MA, USA) and the sonicator was a Sonopuls HD2070 (Bandelin, Berlin, Germany) at ~50% power (~35 W). Luminescence signals (λmax = 559 nm) were recorded by a GloMax^®^-Multi+ Microplate Multimode Reader (Promega, Fitchburg, WI, USA). The GloMax reagent lines and injector were cleaned before and after use with 70% ethanol, then abundantly rinsed with ddH_2_O water.

### 3.3. Synthesis of 6-O-Arachidonoylluciferin (ArLuc-***1***)

To a stirred solution of arachidonic acid (0.18 mmol) in anhydrous CH_2_Cl_2_ (3 mL), under an argon atmosphere and cooled in an ice bath, was added DMAP (2 mg, 0.01 mmol) and 6-hydroxy-2 cyanobenzothiazole (30 mg, 0.18 mmol). DCC (72 mg, 0.35 mmol) was added to the reaction mixture at 0 °C. Stirring was continued overnight at room temperature. Product formation was confirmed by TLC (petroleum ether/ethyl acetate, 95:5). The CH_2_Cl_2_ solution was washed twice with saturated NaHCO_3_ (2.5 mL), dried on anhydrous Na_2_SO_4_, and concentrated to give the crude product as a brown oil. Filtration on column chromatography on silica gel (petroleum ether/ethyl acetate, 95:5) gave 80 mg (0.17 mmol) of the nitrile product as a colorless oil. The nitrile and D-cysteine hydrochloride monohydrate (0.19 mmol) were dissolved in a mixed solvent of 1.5 mL MeOH and 400 μL CH_2_Cl_2_ in an argon atmosphere. A total of 500 μL H_2_O and K_2_CO_3_ (0.19 mmol) were added, and the solution was kept stirring at RT for 1 h. HCl 1M was added until the solution reached a 2–3 pH value. The precipitate was collected and washed with water to give compound ArLuc-**1** as a white solid (30 mg overall yield 30%). ^1^H–NMR (CDCl_3_, 500 MHz): δ (ppm) 0.90 (3H, t, J = 7.6, 20″-CH_3_), 1.28-1.40 (6 H, m, 17″–19″ CH_2_), 1.88 (2H, tt, J = 7.0, 7.6 Hz, 3″-CH_2_), 2.06 (2H, dt, J = 7.6, 7.6 Hz, 16″-CH_2_), 2.24 (2H, dt, J = 7.0, 7.4 Hz, 4″-CH_2_), 2.64 (2H, t, J = 7.0 Hz, 2″-CH_2_), 2.82-2.88 (6H, m, 7″–10″- and 13″-CH_2_), 3.80-3.87 (2H, AB part of ABX system, 5a- and 5b-H), 5.32-5.50 (9H, m, 5″–6″–8″–9″–11″–12″–14″–15″-CH and 4-H), 7.29 (1H, dd, J = 2.3, 8.9 Hz, 5′-H), 7.73 (1H, d, J = 2.3 Hz, 7′-H), 8.16 (1H, d, J = 8.9 Hz, 4′-H). ^13^C–NMR (CDCl_3_, 500 MHz): δ (ppm) 14.1 (20″), 22.6 (19″), 24.7 (3″), 25.7 (7″, 10″, 13″), 26.5 (4″), 27.2 (16″), 29.3 (17″), 31.5 (18″), 33.7 (2″), 35.1 (5), 78.2 (4), 114.7 (7′), 121.6 (5′), 125.2 (4′), 127.5, 127.8, 128.0, 128.4, 128.6, 128.6, 129.3, 130.5 (5″, 6″, 8″, 9″, 11″, 12″, 14″, 15″), 136.7 (8′), 149.7 (6′), 150.8 (9′), 158.0 (2), 160.3 (2′), 167.1 (1″), 171.9 (COOH). IUPAC numbering of 6-O-arachidonoylluciferin, ^1^H and ^13^C NMR spectra of 6-*O*-arachidonoylluciferin (**1**) are reported in Supplementary Materials.

### 3.4. Stability of the Substrate

The stability of ArLuc-**1** was assessed in PLG2 working solution. A total of 10 μL of the ArLuc-**1** working solution were pipetted into the wells of a white 96-well plate; the injection system of the instrument automatically injected 100 µL of PLG2 mix into the well to be analyzed, and the luminescence was read every 30 min for 150 min.

### 3.5. PLG2 Expression and Purification

The pQE-30 vector containing the PLG2 cDNA sequence (GenBank: KY486507) fused with N-terminal 6xHis tag was used to transform *E. coli* JM109 competent cells subsequently grown on LB plates containing ampicillin (100 μg/mL) for selection. PLG2 expression was induced in liquid cultures with 0.1 mM IPTG, overnight at 300 rpm and 37 °C, to avoid protein precipitation. The cells were harvested by ultracentrifugation at 5000× *g* for 10 min and frozen at −80 °C for subsequent purification. Frozen cell pellets were lysed by sonication, and 6xHis-PLG2 was affinity purified on Ni-NTA agarose according to the manufacturer’s instructions. Positive elution fractions were pooled and dialyzed against PCB buffer (50 mM Tris, 150 mM NaCl, 1 mM DTT, 1 mM EDTA, pH 7) using a 3–12 mL-10kDa Slide-A-Lyzer™ Dialysis Cassette. The protein concentrate was divided into small aliquots, flash-frozen into liquid nitrogen, and stored at −80 °C. The purity of purified PLG2 was checked by SDS-PAGE, followed by Coomassie staining.

### 3.6. Stock, Working, and Enzymatic Assay Solutions

LH_2_: LH_2_ working solution was prepared at 0.5 mM concentration by dissolving LH_2_ potassium salt in Tris-HCl 50 mM, pH 7.8. The concentration was adjusted by dilution based on UV using a molar absorptivity coefficient of 7600 M/cm at 266 nm. LH_2_ calibration curve was obtained pipetting appropriate amounts of the 0.5 mM LH_2_ working solution into test tubes containing DMSO and adding 50 mM Tris-HCl pH 7.8 buffer with 1 mM EDTA (DMSO 5%).

ATP: ATP solution was prepared by dissolving 9 mM ATP in 50 mM Tris-HCl, pH 7.8. The concentration was adjusted by dilution based on UV using a molar absorptivity coefficient of 15,400 M/cm at 259 nm.

ArLuc-**1**: The substrate ArLuc-**1** was dissolved in DMSO at 1 mM concentration to obtain stock solution. To prepare working solution an aliquot of stock, solution was diluted 1:20 in DMSO. To prepare ArLuc-**1** testing solutions, ArLuc-**1** stock solution was diluted in DMSO to final concentrations of 1, 10, and 50 μM. To prepare ArLuc-1 reagent solution, ArLuc-**1** working solution was pipetted into an Eppendorf tube, then 50 mM Tris-HCl buffer, pH 7.8, was slowly added, vortexing the tube to obtain homogeneous solution at 2.8 μM final concentration.

PLG2: PLG2 working solution was prepared from an aliquot of purified PLG2, ice-thawed, by dilution to a 3.3 μM concentration with 50 mM Tris-HCl, pH 7.8. MgSO_4_ and ATP solution were added to a final concentration of 10 mM and 0.4 μM, respectively. PLG2 calibration solution for the LH_2_ calibration curve was prepared as PLG2 working solution and DMSO were added to reach a final concentration of 2.5%. PLG2 assay mix solution for the enzymatic assay was prepared from an aliquot of purified PLG2, ice-thawed, by dilution to a 3.3 μM concentration with 50 mM Tris-HCl, pH 7.8. MgSO_4_, ATP, and ArLuc-**1** were added at final concentration of 10 mM, 0.4 μM, and 1.4 μM, respectively.

*h*MAGL: *h*MAGL was diluted to 250 ng/mL in 50 mM Tris-HCl buffer pH 7.8 containing 1mM EDTA.

JZL184: JZL184 stock solution was prepared by dissolving JZL184 powder into DMSO at 4 mM concentration. JZL184 working solutions were prepared at four different concentrations, (40, 20, 4, 0.4 μM) by diluting an aliquot of stock solution with DMSO.

Any leftover mix could be stored at 4 °C and used within 48 h with no loss in performance.

### 3.7. PLG2 DMSO Trials

The DMSO concentration effects on the enzyme activity was determined by adding different amounts of DMSO to PLG2 working solution (0.5%, 1%, 2,5%, and 5% (*v*/*v*)). A sample of PLG2 working solution without DMSO was treated as control. The enzyme stability was expressed as the remaining activity assayed relative to the control value. All measurements were performed as follows: 100 µL of solution containing LH_2_ were pipetted into the wells of a white 96-well plate. The injection system of the instrument would then automatically inject 100 µL of PLG2 mix into the first well to be analyzed. After the injection, the luminometer had a lag time of 1 s, and then integrated the luminescence signal for 1 s. The luminometer would then pass to the following well to be analyzed. The reagent lines and injector were cleaned before and after use with 70% ethanol.

### 3.8. PLG2 ArLuc-***1*** Trials

The effects of ArLuc-**1** concentration on PLG2 enzymatic activity were assessed at 3 different concentrations of ArLuc-**1** (0.1, 1, 5 µM). In a typical experiment, 100 µL of the LH_2_ working solution were pipetted in a well of a white 96-well plate, and 20 µL of the appropriate ArLuc-**1** testing solution were added. Then, the injection system of the instrument automatically injected 100 µL of PLG2 working solution into the first well to be analyzed, and the plate was read every 3 min for 10 min. The time course analyses were linearly regressed, and the slope obtained used to compare PLG2 activity considering as 100% activity the experiment performed without the addition of ArLuc-**1**.

### 3.9. Linearity Tests and Sensitivity of the Method

Calibration curves with LH_2_ standards at different concentrations were built for PLG2. All measurements were performed as described, above (paragraph 3.8.). The tested LH_2_ concentrations were 0.5, 2.5, 5, 12.5, 25, 50, 125, 250 nM. The LOD and LOQ for the assay were calculated on calibration curves. Six blank values were averaged (50 mM Tris-HCl pH 7.8 buffer with 1 mM EDTA, DMSO 2.5%), and the resulting SD was used to calculate LOD and LOQ.

### 3.10. MAGL Bioluminescent Assays and Kinetic Tests

MAGL assays were set up as follows: 10 μL of the *h*MAGL solution (enzyme 25 ng) were pipetted at the bottom of the appropriate well; then, 90 μL of the PLG2 assay mix were quickly added to the enzyme, and the plate was read immediately and every 3 min for 10 min. The kinetic values were determined for *h*MAGL with ArLuc-**1** as substrate. Experiments were set up in the same fashion as the ones above. This time, six reagent solutions were prepared for ArLuc-**1** in-well final concentrations of 0.5, 1, 5, 8, 10, and 20 μM. For negative controls, 50 mM Tris-HCl, pH 7.8 was used, while for blank samples, 50 mM Tris-HCl, pH 7.8 containing 2.5% DMSO was used. The signal coming from negative control solutions was subtracted to that of the corresponding concentration and time point, in order to consider only the signal due to enzymatic hydrolysis. The resulting values were converted into LH_2_ µmoles in accordance with the calibration curves, and the data points were regressed to a linear curve. The slope of each curve was converted into µmol/min/mg produced by the enzyme. The values between the various experiments were averaged and fit to a Michaelis–Menten equation.

### 3.11. MAGL Assay Validation

JZL184 was chosen for the method validation due to its well-known MAGL inhibitory activity. In a typical experiment, 10 μL of the appropriate JZL184 working solution, or of the stock solution for the highest inhibitor concentration or of DMSO for 100% *h*MAGL activity curve, were diluted in an Eppendorf tube with PLG2 working solution that was added dropwise and vortexed to obtain a homogeneous solution, avoiding JZL184 precipitation. *h*MAGL solution was then added and incubated for 30 min. Then, 10 μL of Arluc-**1** working solution were pipetted into a well of a white 96-well plate, and 90 μL of the solution prepared before and incubated were added, and the luminescence assay was run. Final concentrations for JZL184 were 100, 1, 0.5, 0.1, and 0.01 μM. For all the other reagents (*h*MAGL, PLG2, Arluc-**1**), final concentrations resemble that of bioluminescence assays. Blank and negative controls were also analyzed. Control experiment samples were performed as validation experiment without the addition of *h*MAGL. For blank experiments, 10μL of the appropriate JZL184 working solution, or of the stock solution for the highest inhibitor concentration, were diluted in an Eppendorf tube with PLG2 assay buffer (50 mM Tris-HCl, pH 7.8, 10 mM MgSO4, 0.4 μM ATP) that was added dropwise. The tube was vortexed to obtain a homogeneous solution avoiding JZL184 precipitation. After 30 min, 10 μL of Arluc-**1** working solution were pipetted in a well, 90 μL of the appropriate blank solution were added, and the assay was run. In a typical time, course analysis luminescence was read every 3 min for 10 min. The resulting points were regressed to a linear curve (r2 ≥ 0.98 for every curve). The slopes were normalized, and then that of the curve relative to *h*MAGL without inhibitors was taken as the 100% value, and the others were related to this one.

### 3.12. In silico Molecular Docking Simulations

Protein coordinates were downloaded from the Protein Data Bank, accession code 5ZUN. The Schrödinger suite 2017 (Schrödinger, Cambridge, MA, USA) was used for all the computational procedures, and, unless otherwise stated, default settings were applied. The protein was prepared using Protein Preparation Wizard; tetraethylene glycol (PG4), 1,2 ethanediol (EDO), chloride ion (CL), and water molecules (except water molecules HOH542 and HOH585) were removed. Cognate ligand (1-(2-chlorobiphenyl-3-yl)-4-[4-(1,3-thiazol-2-ylcarbonyl)-piperazin-1-yl]pyrrolidin-2-one) and ArLuc-**1** were built using the Maestro Build Toolbar and prepared for docking by Schrödinger Ligand Preparation. The receptor grid was created by the Receptor Grid Generation, the box was centered on the cognate ligand of 5ZUN, and the size of the ligand diameter midpoint cubic box was set to 14 Å. The docking was run with Glide Docking using the extra precision (XP) mode, keeping at most 20 poses per ligand and 200 poses to include in the minimization. The top poses were refined using MM–GBSA (molecular mechanics with generalized Born surface area), as implemented in Prime. Residues at a distance of 12 Å from the ligand were considered as a flexible region in the refinement.

### 3.13. Statistical Analysis

All experiments were performed in triplicate and independently replicated at least once. A calibration curve was constructed to convert luminescence data in to LH_2_ produced. The values of negative controls were subtracted from the enzymatic curve. The initial velocity was determined from the linear portion of the resulting curve. All data were elaborated using GraphPad Prism 6.0c (GraphPad Software, La Jolla, CA, USA), and kinetic parameters Km and Vmax were calculated applying a nonlinear regression analysis (Michaelis–Menten). The quantitative data were calculated as means ± standard errors. The IC50 was calculated with Prism 6 (GraphPad software) using a log (inhibitor) vs. response–Variable slope (four parameters) function.

## 4. Conclusions

Fervent research on the ECS in the last decade has focused on the application of new techniques to deeply investigate the biochemical mechanisms of action of this ubiquitous system. The modulation of the activity of the ECS enzymes remains an important challenge for mechanistic and pharmacological purpose. For this reason, having a range of useful tools for varied research interests such as HTS methods for enzymes inhibitors or activators and development of methods for ex vivo and in vivo assessments is of fundamental importance. Our work, focused on the enzyme MAGL, aimed to add a new useful and very sensitive tool for ECS research. The efficacy of the newly developed luminescence-based assay for measuring the activity of MAGL has been demonstrated. D-luciferin esterified with arachidonic acid, ArLuc-**1**, has been synthesized and was found to enable highly sensitive (LOD 90 pM, LOQ 300 pM) bioluminescent detection of MAGL activity in vitro for potential use in the study of MAGL inhibitors. Recombinant PLG2 was also produced and purified from bacterial cells in good yield (28.7 mg/L). This luciferase variant functioned well in the presence of 2.5% DMSO, confirming its stability and resistance to potentially denaturing conditions. The compatibility of the BL-based MAGL assay with DMSO, which is essential for solubilizing ArLuc-**1**, suggests that additional applications of PLG2 can be developed with reagents that are poorly soluble in aqueous buffers. The BL-assay described here will very likely expand the scope of MAGL inhibitor screening, which often involves solubilization of inhibitors in DMSO. The major advantages of this new bioassay for MAGL activity are high sensitivity and rapidity making it very suitable in applications when the amount of biological sample is limited. ArLuc-**1** represents a valuable addition to the available toolkit of substrates useful for determining the activity of MAGL and can allow the development of efficient HTS protocols for the screening of potential selective inhibitors of this endocannabinoid metabolic enzyme. Furthermore, the very high sensitivity of this method together with the lower biological interference associated with bioluminescence compared to fluorescence-based methods suggests likely applicability to ex vivo or in vivo research.

## Data Availability

Not applicable.

## Supplementary Materials

Supplementary Materials are available online at https://www.mdpi.com/article/10.3390/ijms22116148/s1.
