# Supplementary material for: A New Ultrasensitive Bioluminescence-Based Method for Assaying Monoacylglycerol Lipase
Source: Int J Mol Sci. 2021 Jun 7;22(11):6148. doi: 10.3390/ijms22116148 (PMC8200976; doi:10.3390/ijms22116148)
Supplement: Supplementary file 1 [file ijms-22-06148-s001.zip › ijms-1229027-supplementary.pdf]

Supplemental Data

# A new ultrasensitive bioluminescence-based method for assaying Monoacylglycerol lipase

Matteo Miceli[a], Silvana Casati[a], Pietro Allevi[b], Silvia Berra[a], Bruce R. Branchini[c], Roberta Ottria[a], Paola Rota[b], and Pierangela Ciuffreda[a*]

a. *Dipartimento di Scienze Biomediche e Cliniche "Luigi Sacco", Università degli Studi di Milano, Via G.B. Grassi 74, 20157 Milano, Italy.*
b. *Dipartimento di Scienze Biomediche, Chirurgiche e Odontoiatriche, Dipartimento di Scienze Biomediche, Università degli Studi di Milano, Via della Commenda 10, 20122 Milano, Italy.*
c. *Department of Chemistry, Connecticut College, New London, CT 06320, United States*

**$^1$H and $^{13}$C-NMR data**

*Instruments*

$^1$H-NMR spectra were recorded in $CDCl_3$ (isotopic enrichment 99.95%) solutions at 300 K using a Bruker AVANCE 500 instrument (500.13 MHz for $^1$H, 125.76 MHz for $^{13}$C) using 5 mm inverse detection broadband probes and deuterium lock. Chemical shifts (d) are given as parts per million relative to the residual solvent peak (7.26 ppm for $^1$H and 77.0, central line, for $^{13}$C) and coupling constants ($J$) are in Hertz. The experimental error in the measured $^1$H-$^1$H coupling constants is ± 0.5 Hz. The splitting pattern abbreviations are as follows: s, singlet; d, doublet; t, triplet; q, quartet; m, multiplet, and bs, broad peak. For two-dimensional experiments, Bruker microprograms using gradient selection (gs) were applied. Acquisition parameters for 1D were as follows: $^1$H spectral width of 5000 Hz and 32 K data points providing a digital resolution of ca. 0.305 Hz per point, relaxation delay 2 s; $^{13}$C spectral width of 29412 Hz and 64 K data points providing a digital resolution of ca. 0.898 Hz per point, relaxation delay 2.5 s.

For two-dimensional experiments, standard Bruker microprograms using gradient selection (gs) were applied. COSY-45 experiments were acquired with 512 t1 increments; 2048 t2 points; spectral/spectrum width 10.0 ppm. The acquisition data for HSQC and HMBC experiments were obtained with 512 t1 increments; 2048 t2 points; spectral/spectrum width 10.0 ppm for 1H and 220 ppm for 13C. Delay values were optimized for $^1J_{C,H}$ 140.0 Hz and $^1J_{C,H}$ 3.0 Hz. Zero filling in F1 to 1 K, $\pi$/2 shifted sine-bell squared (for gHSQC) or sinebell (for gHMBC) apodization functions were used for processing.

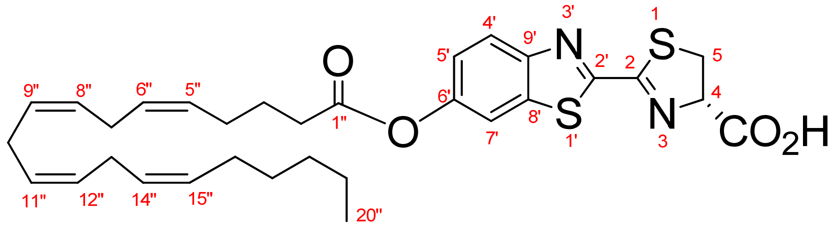


**Figure S1.** IUPAC numbering of 6-*O*-arachidonoylluciferin

$^1$H spectrum of 6-*O*-arachidonoylluciferin (**1**)

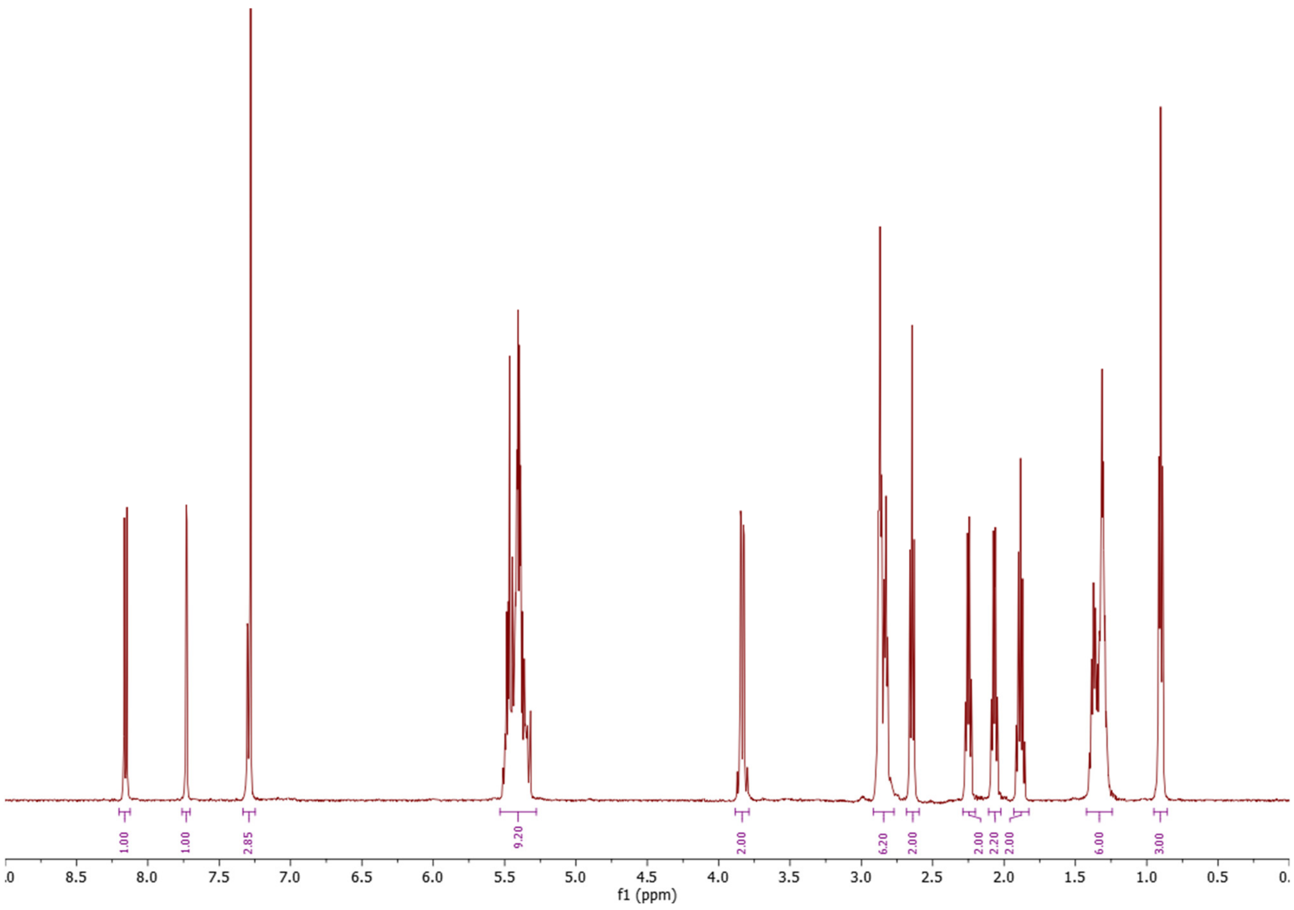

1.00
1.00
2.85
9.20
2.00
6.20
2.00
2.00
2.20
2.00
6.00
3.00
f1 (ppm)


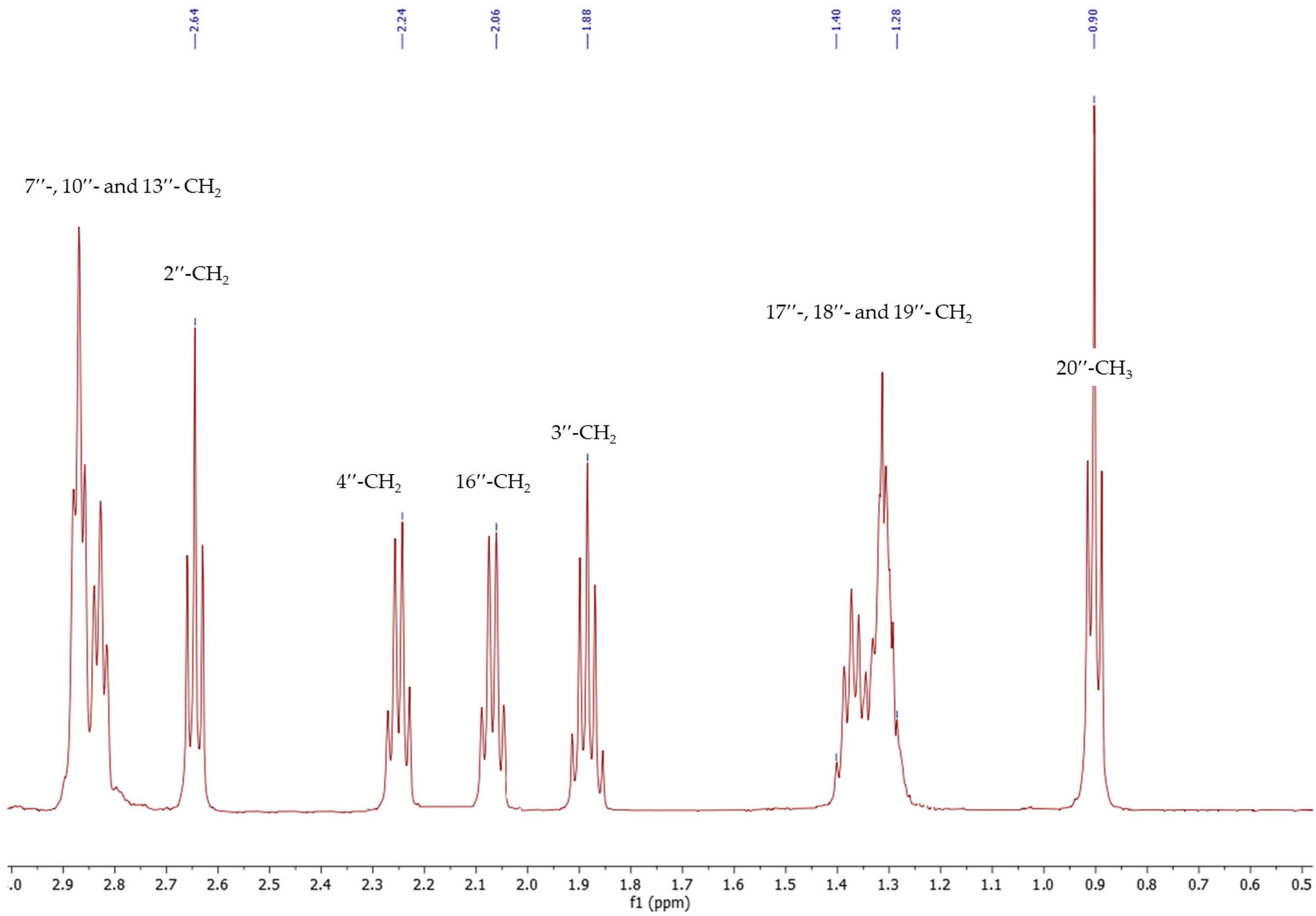

2.64
2.24
2.06
1.88
1.40
1.28
0.90
7′′-, 10′′- and 13′′- CH$_2$
2′′-CH$_2$
17′′-, 18′′- and 19′′- CH$_2$
20′′-CH$_3$
3′′-CH$_2$
4′′-CH$_2$
16′′-CH$_2$
f1 (ppm)

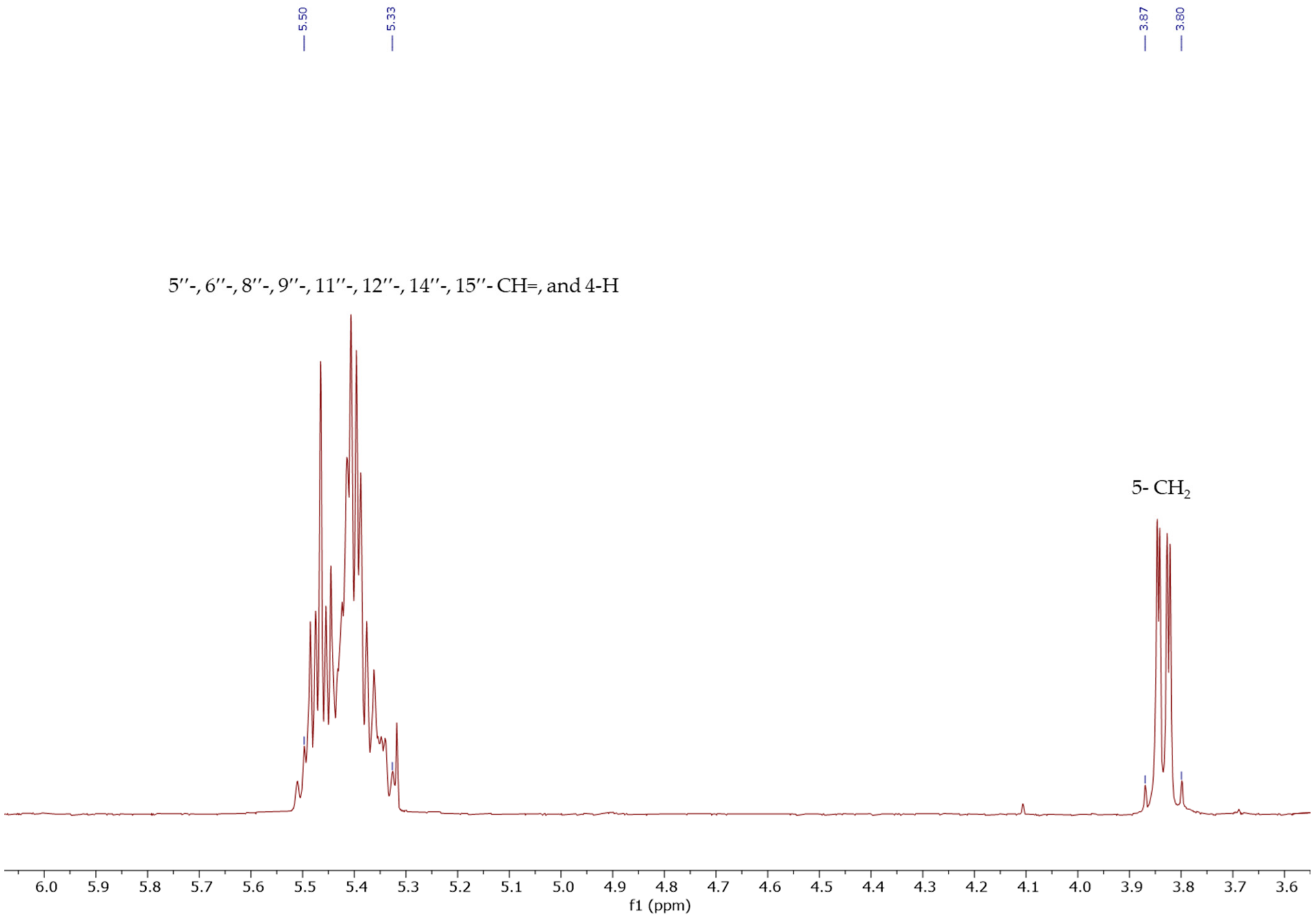
5.50
5.33
3.87
3.80
5′′-, 6′′-, 8′′-, 9′′-, 11′′-, 12′′-, 14′′-, 15′′- CH=, and 4-H
5- CH$_2$
6.0
5.9
5.8
5.7
5.6
5.5
5.4
5.3
5.2
5.1
5.0
4.9
4.8
4.7
4.6
4.5
4.4
4.3
4.2
4.1
4.0
3.9
3.8
3.7
3.6
f1 (ppm)

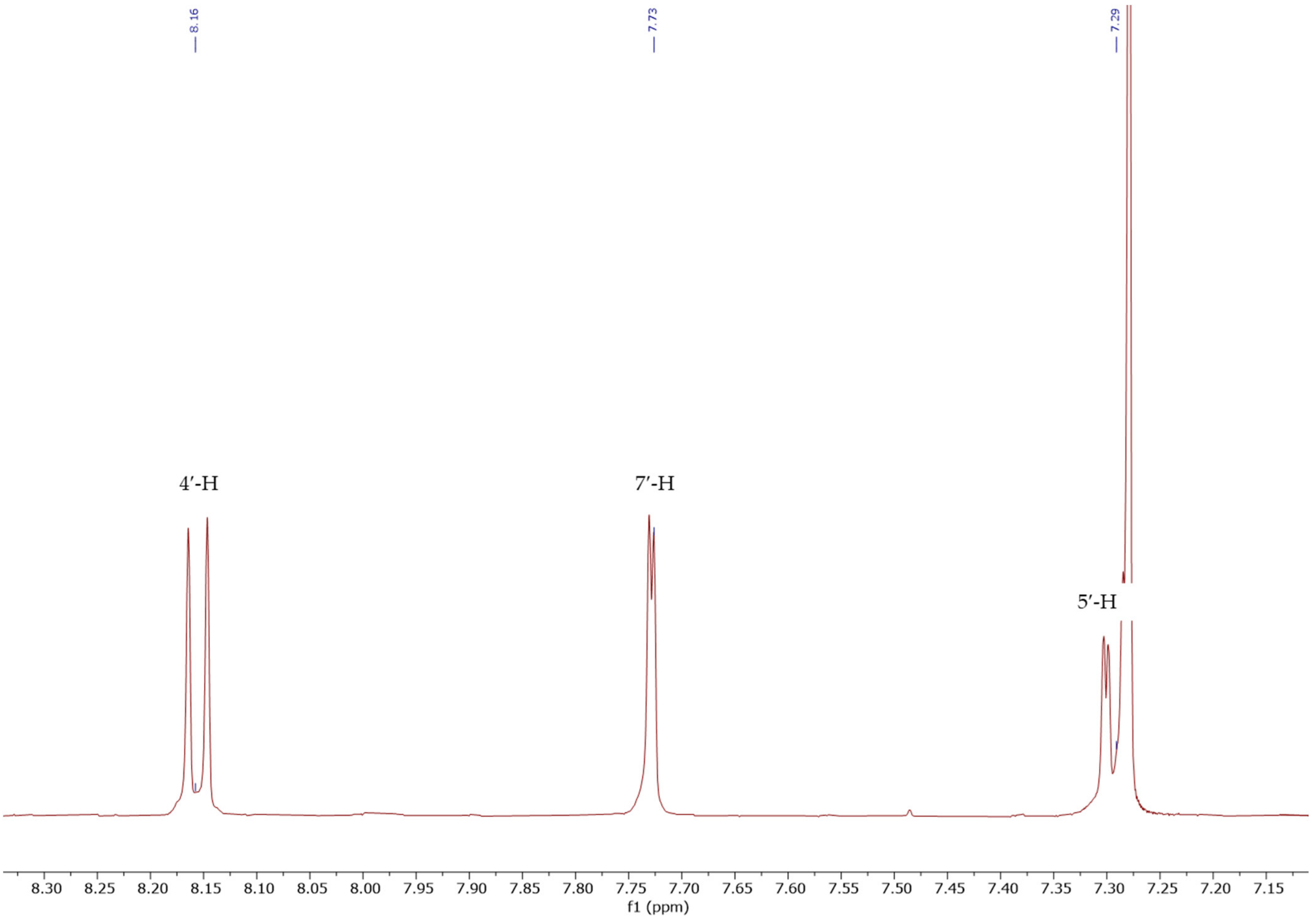
8.16
7.73
7.29
4′-H
7′-H
5′-H
8.30
8.25
8.20
8.15
8.10
8.05
8.00
7.95
7.90
7.85
7.80
7.75
7.70
7.65
7.60
7.55
7.50
7.45
7.40
7.35
7.30
7.25
7.20
7.15
f1 (ppm)

$^1$H NMR ($CDCl_3$, 500 MHz): δ (ppm) 0.90 (3H, t, J=7.6, 20′′-$CH_3$), 1.28-1.40 (6 H, m, 17′′-, 18′′-, 19′′- $CH_2$), 1.88 (2H, tt, J=7.0, 7.6 Hz, 3′′-$CH_2$), 2.06 (2H, dt, J=7.6, 7.6 Hz, 16′′-$CH_2$), 2.24 (2H, dt, J=7.0, 7.4 Hz, 4′′-$CH_2$), 2.64 (2H, t, J=7.0 Hz, 2′′-$CH_2$), 2.82-2.88 (6H, m, 7′′- 10′′- and 13′′-$CH_2$), 3.80-3.87 (2H, AB part of ABX system, 5a- and 5b-H), 5.32-5.50 (9H, m, 5′′- 6′′- 8′′- 9′′- 11′′- 12′′- 14′′- 15′′-CH and 4-H), 7.29 (1H, dd, J=2.3, 8.9 Hz, 5′-H), 7.73 (1H, d, J=2.3 Hz, 7′-H), 8.16 (1H, d, J=8.9 Hz, 4′-H).

$^{13}C$ NMR spectrum of 6-*O*-arachidonoylluciferin (**1**)

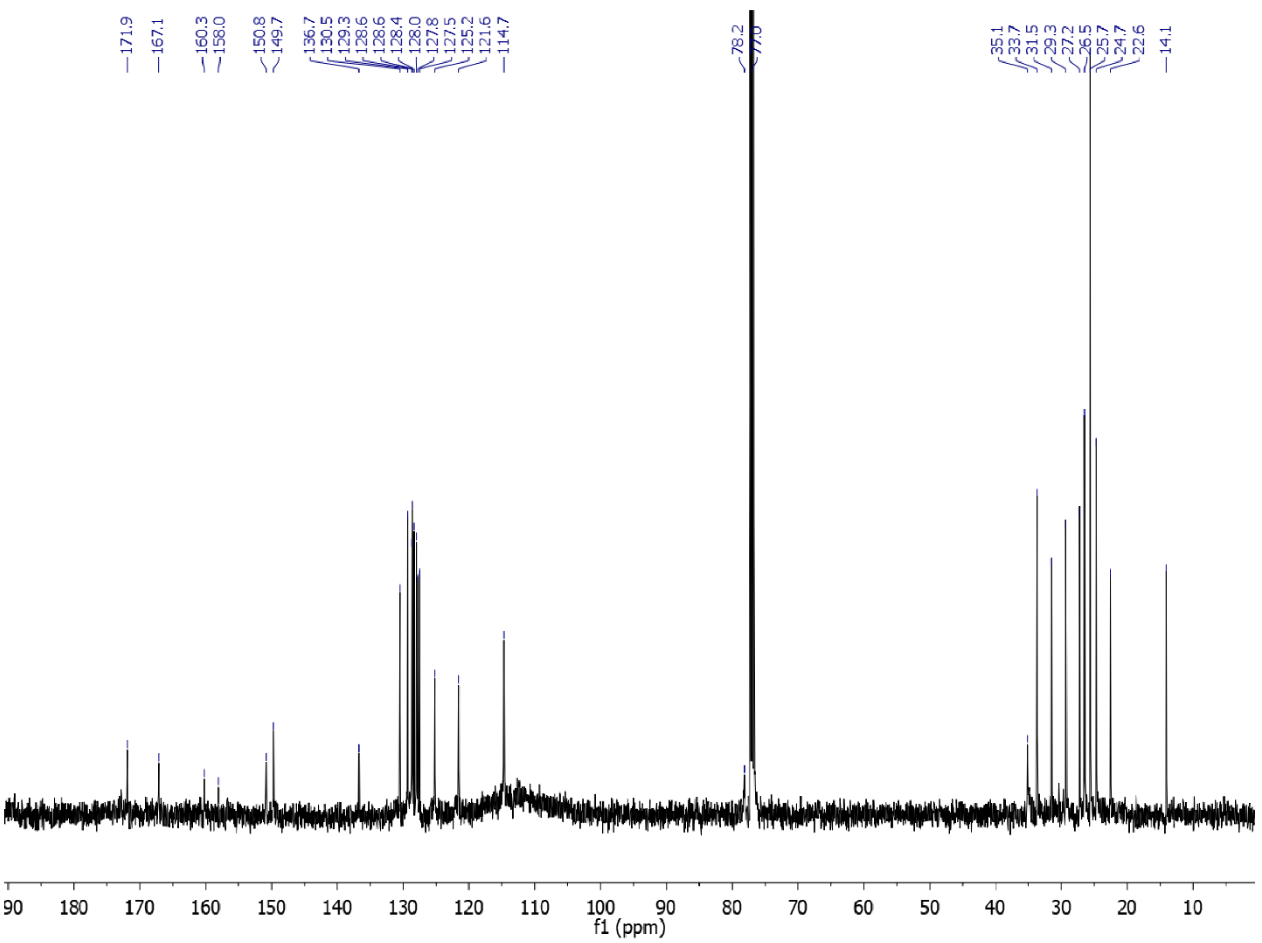


$^{13}C$ NMR ($CDCl_3$, 125.76 MHz): δ (ppm) 14.1 (20′′), 22.6 (19′′), 24.7 (3′′), 25.7 (7′′, 10′′, 13′′), 26.5 (4′′), 27.2 (16′′), 29.3 (17′′), 31.5 (18′′), 33.7 (2′′), 35.1 (5), 78.2 (4), 114.7 (7′), 121.6 (5), 125.2 (4), 127.5, 127.8, 128.0, 128.4, 128.6, 128.6, 129.3, 130.5 (5′, 6′, 8′, 9′, 11′, 12′, 14′, 15′), 136.7 (8′), 149.7 (6′), 150.8 (9′), 158.0 (2), 160.3 (2′), 167.1(1′′), 171.9 (*C*OOH).
